# Performance Evaluation of SpliceAI for the Prediction of Splicing of *NF1* Variants

**DOI:** 10.3390/genes12091308

**Published:** 2021-08-25

**Authors:** Changhee Ha, Jong-Won Kim, Ja-Hyun Jang

**Affiliations:** Department of Laboratory Medicine and Genetics, Samsung Medical Center, Sungkyunkwan University School of Medicine, 81 Irwon-ro, Gangnam-gu, Seoul 06351, Korea; chha8808@naver.com (C.H.); kimjw@skku.edu (J.-W.K.)

**Keywords:** neurofibromatosis type 1, *NF1*, SpliceAI, *in silico* prediction, splice variants

## Abstract

Neurofibromatosis type 1, characterized by neurofibromas and café-au-lait macules, is one of the most common genetic disorders caused by pathogenic *NF1* variants. Because of the high proportion of splicing mutations in *NF1*, identifying variants that alter splicing may be an essential issue for laboratories. Here, we investigated the sensitivity and specificity of SpliceAI, a recently introduced *in silico* splicing prediction algorithm in conjunction with other *in silico* tools. We evaluated 285 *NF1* variants identified from 653 patients. The effect on variants on splicing alteration was confirmed by complementary DNA sequencing followed by genomic DNA sequencing. For *in silico* prediction of splicing effects, we used SpliceAI, MaxEntScan (MES), and Splice Site Finder-like (SSF). The sensitivity and specificity of SpliceAI were 94.5% and 94.3%, respectively, with a cut-off value of Δ Score > 0.22. The area under the curve of SpliceAI was 0.975 (*p* < 0.0001). Combined analysis of MES/SSF showed a sensitivity of 83.6% and specificity of 82.5%. The concordance rate between SpliceAI and MES/SSF was 84.2%. SpliceAI showed better performance for the prediction of splicing alteration for *NF1* variants compared with MES/SSF. As a convenient web-based tool, SpliceAI may be helpful in clinical laboratories conducting DNA-based *NF1* sequencing.

## 1. Introduction

Neurofibromatosis type 1 (NF1; OMIM # 162200) is an autosomal dominant inherited disease and one of the most common human genetic disorders, with an incidence of ~1 in 3000 [1]. NF1 is caused by loss-of-function variants in the tumor suppressor gene, *neurofibromin* 1 (*NF1*; MIM * 613113) [1,2], which is located at chromosome 17q11.2 and contains 60 translated exons spanning over 280 kb of genomic DNA (gDNA) [3,4].

High proportions of the reported *NF1* disease-causing variants are single nucleotide variants (SNVs), small insertions and/or deletions of nucleotides (INDELs) (see Human Gene Mutation Database: http://www.hgmd.cf.ac.uk/, accessed on 1 July 2021) [5], which are predicted to result in a premature termination codon. Notably, the frequency of *NF1* splice variants resulting in aberrant mRNA splicing is significantly higher than that of mutated genes in other genetic diseases [6,7,8]. To achieve a sufficient detection rate of pathogenic variants, a multistep sequence analysis procedure for both *NF1* gDNA and complementary DNA (cDNA) has been recommended [3,6,9].

However, the analysis of *NF1* variants is challenging and burdensome because of the large size of the gene, the presence of several homologous pseudogenes, and a wide mutational spectrum with a lack of mutational hot-spots [6,10,11]. In addition, since mRNA is vulnerable to decay [12,13], the yield, purity, and integrity of extracted mRNA may not be sufficient for cDNA sequence analysis. These obstacles may lead to challenges in identifying splicing variants of *NF1*. When relying on a single technique, variant detection rates are approximately 50 to 80% [7,11], compared with 95% in a combined analysis of gDNA and cDNA [6]. To compensate for the relatively low detection rate of sequencing of only gDNA, predicting the splice effect of *NF1* using *in silico* tools would be beneficial.

Although several algorithms are available for splicing prediction, the sensitivity and specificity of these algorithms are not satisfactory. Recently a novel deep residual neural network tool, SpliceAI, was developed and showed a notable performance for predicting splicing altering effects variants [14]. In contrast to other *in silico* tools that only examine short nucleotide windows adjacent to exon-intron boundaries, SpliceAI learns splicing determinants directly from the primary sequence by evaluating 10,000 nucleotides of the flanking sequence [14]. However, evaluation of the use SpliceAI for *NF1* variants has not been reported. Herein, we investigated the optimal cut-off value for the SpliceAI score using patient data and compared the performance of SpliceAI with other *in silico* tools for the prediction of splicing aberrations in *NF1*.

## 2. Results

### 2.1. Characteristics of Variants

**Figure 1:**
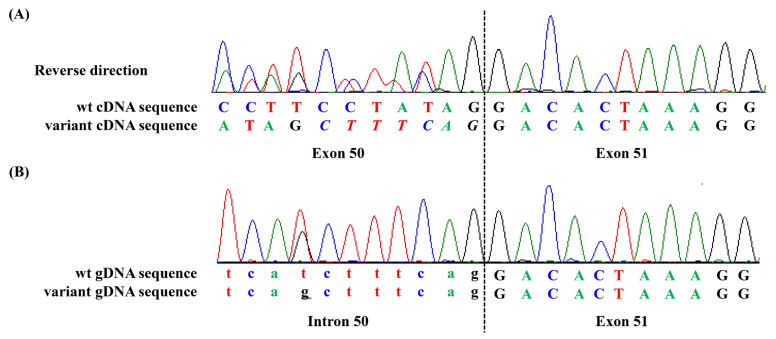
Electrophoretogram of the (**A**) complementary DNA (cDNA) and (**B**) genomic DNA (gDNA) from novel splice variant c.7458-8T>G, resulting in creation of a new 3′ splice site that leads to a 7-nt insertion of c.7458-7_7458-1. Below, cDNA and gDNA sequences of wild type (wt) and splice variant are presented. In the cDNA sequences, the inserted nucleotides are italicized. In the gDNA sequences, the nucleotide substitution is underlined. Small letters indicate the intronic sequence.

A total of 285 unique *NF1* variants were analyzed. Characteristics of the variants are listed in Table 1. Among the variants, 73 were confirmed to result in splicing alteration by cDNA and gDNA sequencing analysis. Confirmed splicing variants were mostly located in canonical splice sites; type I splice variants causing exon skipping [8] were the most common consequence of the splicing effects. One example of splice variants is shown in Figure 1. This variant (c.7458-8T>G) had a Δ Score of 1.00 and correctly matched with splice defect.

Among the confirmed non-splicing variants, SNVs including nonsense, missense, and synonymous variations were most commonly observed. The distribution of SpliceAI Δ Scores was notably different between confirmed splicing and non-splicing variants (Table 1). Of the 285 *NF1* variants in this study, 52 were novel and 9 of them were splicing variants. More detailed information about the variants in this study is provided in supplementary information (Table S1).

### 2.2. Sensitivity and Specificity

Since few reports have examined the cut-off value for Δ Score of SpliceAI, receiver operating characteristic (ROC) curve analysis was performed [15]. Based on this analysis, the optimal cut-off was determined to be >0.22 with an area under the ROC curve (AUC) of 0.975 (*p* < 0.0001, area = 0.5). Under this value, the sensitivity and specificity were determined as 94.5% (95% confidence interval (CI), 86.6–98.5%) and 94.3% (95% CI, 90.3–97.0%), respectively. The range of Δ Score of false negative and false positive was 0.00–0.13 and 0.25–1.00, respectively (Table S1). Among 30 confirmed splicing variants located in the non-canonical intronic regions, 100% were accurately predicted by SpliceAI.

By the combined analysis of MaxEntScan (MES) [16] and Splice Site Finder-like (SSF) [17]; abbreviated as MES/SSF further in the manuscript, the sensitivity and specificity were 83.6% (95% CI, 73.1–91.2%) and 82.6% (95% CI, 76.8–87.4), respectively (Table 2). SpliceAI had a slightly higher sensitivity (difference 11.0%, *p* = 0.0636) and significantly higher specificity (difference 11.8%, *p* = 0.0003) compared with MES/SSF.

### 2.3. Pairwise Comparison of Receiver Operating Characteristic Curves

**Figure 2:**
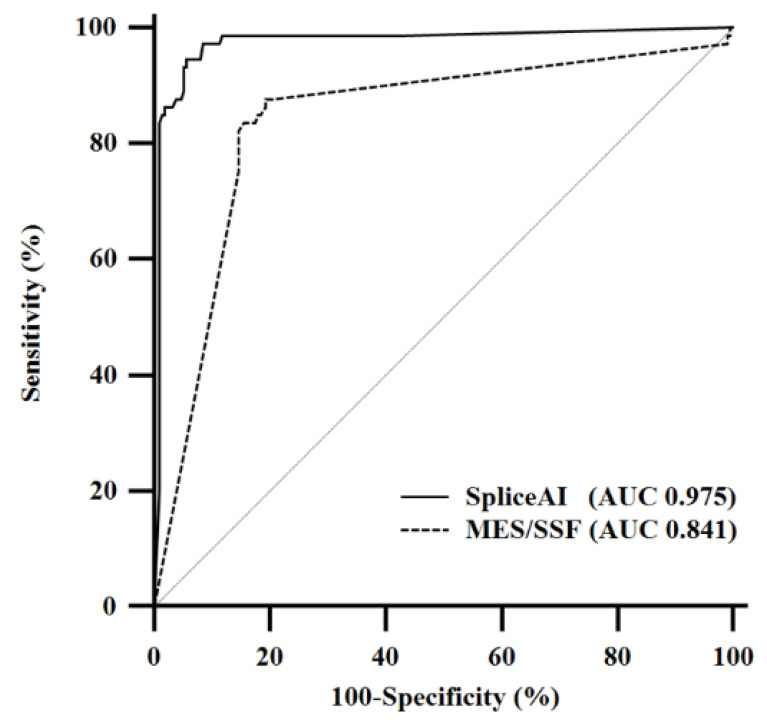
Receiver operating characteristic curves and an area under the receiver operating characteristic curve comparing SpliceAI and MES/SSF for predicting the splice effect of 285 *NF1* variants. Abbreviations: MES, MaxEntScan; SSF, splice site finder-like; AUC, area under the receiver operating characteristic curve.

The AUC of MES/SSF was 0.841 (*p* < 0.0001, area = 0.5); however, SpliceAI showed a significantly larger AUC (difference 0.134, *p* < 0.0001). The ROC curves of SpliceAI and MES/SSF are shown in Figure 2.

### 2.4. Concordance Rate

Among the 285 unique variants, 84.2% were concordant between SpliceAI and MES/SSF. The calculated positive percent agreement (PPA), negative percent agreement (NPA), and kappa value were 68.4% (95% CI, 58.6–76.7), 92.5% (95% CI, 87.8–95.5), and 0.64 (95% CI, 0.54–0.73), respectively (Table 3).

Forty-five discordant variants between SpliceAI and MES/SSF were mainly located in exons. All 31 variants with SpliceAI (−) and MES/SSF (+) were confirmed to be splicing negative by cDNA and gDNA sequencing analysis. Among the remaining 14 variants with SpliceAI (+) and MES/SSF (−), 8 variants were confirmed to be splicing positive (Table 4).

## 3. Discussion

*NF1* has a distinctive feature that the proportion of splicing variants is relatively high, accounting for 22–30% of pathogenic variants (https://www.ncbi.nlm.nih.gov/books/NBK1109/, accessed on 15 July 2021). Deep-intronic or synonymous variants, even missense or nonsense variants, can result in splicing alterations and most may be classified as variants of uncertain significance without cDNA sequence analysis. For this reason, a multistep approach based on cDNA and gDNA sequence analysis could improve the diagnostic yield [3,6,9]. If cDNA sequence analysis is performed for only splicing positive cases by *in silico* analysis, the sensitivity and specificity of the *in silico* tools affect diagnostic yield and laboratory workload. Earlier studies evaluating *in silico* splicing tools were mainly based on the analysis of variants in multiple genes, including *BRCA1/BRCA2* or *FBN1*, or a small number of variants in a single gene such as *RB1* and *LDLR* [18,19,20,21]. To the best of our knowledge, our study is the largest *in silico* study of *NF1* and examined 285 unique *NF1* variants identified from more than 600 independent patients.

Few reports have investigated the prediction power of SpliceAI using clinical data. In one study evaluating 257 variants, which included 33% aberrant splicing variants confirmed by cDNA sequence analysis, SpliceAI showed 89.9% sensitivity and 91.6% specificity with a cut-off value of 0.2 [22]. These results were similar with those of the present study, showing 94.5% sensitivity and 94.3% specificity with a cut-off value of >0.22. The present study might be highlighted in that a large number of variants of *NF1* were evaluated, since previous studies using SpliceAI evaluated mainly variants of *BRCA1/BRCA2*, *CFTR*, *FBN1*, and *PLCγ1* genes [22,23,24].

In a study comparing *in silico* splicing prediction tools, SpliceAI showed better performance than other tools [22]. In the present study, the prediction power of SpliceAI was better than the combined analysis of MES/SSF. The difference in the performance of splicing variant prediction between MES/SSF and SpliceAI would probably be due to the regional differences used in algorithm training. Most *in silico* splicing prediction tools analyze SNVs [25] located near the exon-intron junction or splicing consensus regions (e.g., Cartegni region; see Methods) [19,25]. On the other hand, SpliceAI has the ability to predict splice effects on a wide-spectrum of variant positions [14], not limited to the splicing consensus regions. This is possible since SpliceAI was developed by training pre-mRNA transcript sequences and whole-genome sequencing data [14,26,27,28,29]. The major proportion of the discrepant prediction between SpliceAI and MES/SSF were variants located in exons (Table 4), with 31 variants were falsely predicted by MES/SSF, proven by cDNA and gDNA sequencing analysis. Since they were mostly deep exon variants, ranging 6–213 bp to the original splice site, prediction of MES/SSF would not be properly made and SpliceAI showing better performance is reasonable. In the present study, SpliceAI precisely predicted deep exonic splice variants, c.1466A>G and c.3304T>G (Δ score of 0.99 and 1.00, respectively). Deep intronic splice variants including c.288+1137C>T, c.1260+1604A>G, and c.5610-456G>T (0.72, 0.76, and 0.93, respectively) were also well predicted. Another study reported a deep intronic splice variant c.1392+754T>G [30], and SpliceAI predicted well with a Δ score of 0.72.

There have been some difficulties for laboratories to use *in silico* splicing prediction tools since several tools are available however, there is no consensus cut-off value. For MES [16], cut-off values of 10%, 15%, and 20% have been suggested [18,31,32]. In contrast, 5% was used for NNSplice and SSF and 2% was used for Human Splicing Finder [18,31,33,34]. Furthermore, when multiple tools are used for better prediction, the definition of “positive” prediction would be more complicated. In one study, positivity was indicated when two out of three *in silico* tools were in agreement, whereas another study determined positivity when three out of four *in silico* tools agreed [22,35]. In this regard, SpliceAI as a single tool of outperforming performance could be useful for predicting splice variants.

In addition to the prediction power, SpliceAI has advantages in that it can be assessed online (v1.3.1, https://spliceailookup.broadinstitute.org/#, accessed on 1 July 2021) [14]. Data input is more intuitive for SpliceAI compared with other *in silico* splicing prediction tools since genomic position or the Human Genome Variation Society (HGVS) nomenclature can be used instead of the FASTA format. However, the consensus cut-off values remain to be determined. Although the present study used the cut-off value of Δ score > 0.22 through the ROC analysis, previous studies reported a range of values, from 0.2 to 0.85, depending on genes and variant sites [22,24,36,37]. Since the optimal cut-off value might differ by genes and/or location of the variants within a gene, validation studies using an RNA-confirmed clinical dataset are required.

## 4. Materials and Methods

### 4.1. Study Subjects

**Figure 3:**
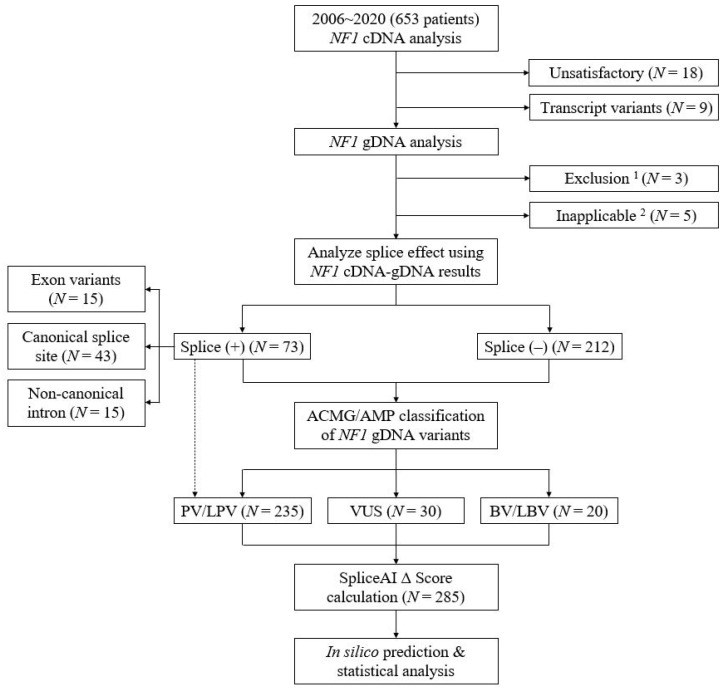
Study workflow. ^1^ Genomic DNA analysis showed no relevant variants. ^2^ Variants other than single nucleotide polymorphisms and simple insertions and/or deletions of bases. N denotes different types of *NF1* variants. Abbreviations: cDNA, complementary DNA; gDNA, genomic DNA; ACMG, American College of Medical Genetics and Genomics; AMP, Association for Molecular Pathology; PV, pathogenic variant; LPV, likely pathogenic variant; VUS, variant of uncertain significance; BV, benign variant; LBV, likely benign variant.

We retrospectively analyzed the gDNA and cDNA variants identified from 653 patients tested for *NF1* sequencing between January 2006 and December 2020. In accordance with the American College of Medical Genetics and Genomics/Association for Molecular Pathology guideline [38], *NF1* variants were classified into three categories (Table S1): (1) pathogenic variant (PV)/likely PV (LPV), (2) variant of uncertain significance (VUS), and (3) benign variant (BV)/likely BV (LBV). During categorization, allele frequencies were reviewed using gnomAD (v2.1.1, https://gnomad.broadinstitute.org/, accessed on 1 July 2021). Previous reports of *NF1* variants were reviewed using Human Genome Variation Database (HGMD^®^ Professional release 2021.2, https://my.qiagendigitalinsights.com/bbp/view/hgmd/pro/start.php, accessed on 1 July 2021), ClinVar (https://www.ncbi.nlm.nih.gov/clinvar/, accessed on 1 July 2021), and Leiden Open Variation Database (LOVD; https://databases.lovd.nl/shared/variants/NF1, accessed on 1 July 2021). Functional study was performed using cDNA and gDNA sequencing analysis (see Section 4.2). The following *NF1* cDNA or gDNA variants were excluded: (1) variants with unsatisfactory quality or insufficient variant information in cDNA and/or gDNA sequencing analysis, (2) benign mRNA transcripts in RT-PCR, and (3) mRNA variants with no identifiable corresponding gDNA variant, and (4) gDNA variants for which SpliceAI Δ Score could not be obtained in the range of 0–1 (variants other than SNVs or simple INDELs, see Section 4.3). Our study workflow and the number of excluded cDNA/gDNA variants are shown in Figure 3. Based on the exclusion criteria above, 285 unique gDNA variants of *NF1* were included for the evaluation, including 73 splice variants (proven by cDNA and/or gDNA sequencing analysis). Among the 285 variants, 235, 30, and 20 were PVs/LPVs (including all 73 splice variants), VUSs, and BVs/LBVs, respectively (Figure 3). This study was approved by the Institutional Review Board of Samsung Medical Center, Seoul, Korea (protocol code 2021-05-122, approved on 6 July 2021).

### 4.2. Complementary DNA and Genomic DNA Sequencing

Peripheral blood samples were collected in a vacuum tube containing ethylenediaminetetraacetic acid as a preservative and gDNA and RNA were extracted from leukocytes. DNA was extracted using a Wizard Genomic DNA Purification Kit (Promega, Madison, WI, USA) according to the manufacturer’s instructions. The concentration and purity of the DNA were measured using NanoDrop (Thermo Fisher Scientific, Waltham, MA, USA). The RNA was extracted with TRIzol methods and 1 µg of samples were reverse transcribed using Thermo Scientific RevertAid First Strand cDNA Synthesis Kit (Thermo Fisher Scientific, Waltham, MA, USA). RT-PCR and cDNA sequencing were performed to screen altered splicing and coding region variants of *NF1*. Amplification of *NF1* cDNA was performed through 24 overlapping fragments using the GeneAmp PCR System 9700 Thermal Cycler (Applied Biosystems, Foster City, CA, USA). The primer sets for gDNA and cDNA amplification were based on previous report from our institution [30], which are listed in Supplementary Tables S2 and S3, respectively. Cyclic sequencing was performed using the BigDye Terminator v3.1 Cycle Sequencing Kit (Applied Biosystems), and sequence traces were obtained on an ABI 3730xl DNA Analyzer (Applied Biosystems). Sequence variations were detected through Sequencher software (Gene Codes, Ann Arbor, MI, USA). If a variant or exon skipping was detected in the cDNA sequence analysis, the involved exon and flanking intronic regions of gDNA were sequenced using gDNA to identify the corresponding DNA variant that caused splicing alterations. The reference sequence for alignment and variant detection was based on NM_001042492.2, the longest isoform for *NF1*. Variants were described according to the HGVS guidelines (http://varnomen.hgvs.org/, accessed on 1 July 2021) [39].

### 4.3. Splicing Prediction

SpliceAI, a web-based interface (https://spliceailookup.broadinstitute.org/#, accessed on 1 July 2021), was used for splicing prediction. The Δ Score, the maximum probability of splicing where a variant affects the gain or loss of acceptor or donor sites, was obtained for each variant using default parameters. The Δ Scores could be obtained only in SNVs and *simple* INDELs; reference or alternative allele in the reference genome is a single base. More detailed information on the Δ Score has been previously described [14].

For the comparison of the performance of the SpliceAI with other *in silico* splicing prediction tools, MES [16] and SSF [17], two commonly used algorithms, were conducted using Alamut^®^ Visual v.2.15 software (SOPHiA GENETICS, Saint-Sulpice, Switzerland). According to the previous recommendations [18,40], variants were considered as positive for splicing alteration based on the following: (1) MES predicted >15% reduction of the score of the natural splice site AND SSF predicted >5%, or (2) a new splice site was created. If a variant was not placed within the Cartegni region (i.e., 11 bases for the 5′ splice site; from the last 3 exonic to the first 8 intronic bases, and 14 bases for the 3’ splice site; from the last 12 intronic to the first 2 exonic bases) [41], we only applied SSF prediction as mentioned above.

### 4.4. Statistical Analysis

Using the Δ Scores from SpliceAI, ROC curve analysis was conducted based on the method developed by Hanley and McNeil [15]. The optimal cut-off value of Δ Scores was obtained from ROC curve analysis. In further analysis, a single *NF1* variant was defined as SpliceAI prediction “positive” when the SpliceAI Δ Score of the variant was above the cut-off value.

PPA, NPA, kappa coefficient, and their 95% CIs were calculated to compare the agreement between SpliceAI and other *in silico* tools (MES/SSF) for predicting the splice effect of the variants. The sensitivity and specificity of SpliceAI and MES/SSF were calculated against the splicing effect using cDNA and gDNA sequencing analysis results. McNemar’s test was used to compare the sensitivity and specificity between SpliceAI and MES/SSF for splice prediction. Using Δ Scores (SpliceAI) and reduction ratio (MES/SSF), pairwise comparison of ROC curves between SpliceAI and MES/SSF were also performed. Statistical analyses were performed using MedCalc Statistical Software version 19.0.5 (MedCalc Software, Ostend, Belgium). *p* < 0.05 was considered statistically significant.

## 5. Conclusions

This is the largest single-center study on evaluating the use of SpliceAI in an *in silico* study on *NF1* variants, comparing the actual functional effect of a variant through cDNA and gDNA sequencing analysis as well as other *in silico* tools (MES/SSF). Our data indicated that SpliceAI showed moderate agreement with MES/SSF, and outperformed MES/SSF in terms of sensitivity and specificity. Our observations indicate that SpliceAI is a convenient and effective *in silico* splicing prediction tool. These results suggest the potential for SpliceAI in predicting variants in addition to *NF1* in routine genetic laboratories due to its convenience and predictive value.

## Figures and Tables

**Table 1:** Classification and description of the identified *NF1* variants.

Variant Classification	Number of Different Variants	SpliceAI Δ Score ^1^
Total variants	285	0.01 (0.00–0.36)
Splice variants	73	0.98 (0.80–0.99)
Variant location		
Canonical splice-site	43	0.99 (0.95–1.00)
Non-canonical intronic region	15	0.91 (0.55–0.98)
Exon	15	0.54 (0.13–0.99)
Splicing classification ^2^		
Type I	35	0.97 (0.87–1.00)
Type II	3	0.76 (0.72–0.93)
Type III	5	0.99 (0.97–1.00)
Type IV	20	0.99 (0.98–1.00)
Type V	10	0.30 (0.12–0.62)
Non-splice variants	212	0.00 (0.00–0.02)
Frameshift	68	0.00 (0.00–0.04)
Nonsense	68	0.01 (0.00–0.03)
Missense	48	0.00 (0.00–0.01)
Synonymous	22	0.00 (0.00–0.01)
In-frame deletion	5	0.00 (0.00–0.01)
Start loss	1	0.00 (0.00–0.00)

^1^ Values expressed as median (25th percentile–75th percentile). ^2^ Classification system of *NF1* splicing mutations by Wimmer et al. [8]; exon skipping from variants at authentic splice sites (type I), cryptic exon inclusion caused by deep intronic variations (type II), creation of *de novo* splice sites causing loss of exonic sequences (type III), activation of cryptic splice sites upon authentic splice-site disruption (type IV), and exonic sequence alterations causing exon skipping (type V).

**Table 2:** Performance of SpliceAI and MES/SSF for predicting *NF1* splice effect.

Method	Sensitivity	Specificity
	N/Total N% (95% CI)	N/Total N% (95% CI)
SpliceAI	69/73	200/212
	94.5% (86.6–98.5%)	94.3% (90.3–97.0%)
MES/SSF	61/73	175/212
	83.6% (73.1–91.2%)	82.5% (76.8–87.4%)

Abbreviations: CI, confidence interval; MES, MaxEntScan; SSF, Splice-Site Finder-like.

**Table 3:** Agreement analysis of SpliceAI and MES/SSF for predicting *NF1* splice effect.

Method			MES/SSF	
		Positive	Negative	Total
	Positive	67	14	80
SpliceAI	Negative	31	173	205
	Total	98	187	285
	Positive percent agreement = 68.4% (95% CI, 58.6–76.7) Negative percent agreement = 92.5% (95% CI, 87.8–95.5) Kappa value = 0.64 (95% CI, 0.54–0.73)			

Abbreviations: CI, confidence interval; MES, MaxEntScan; SSF, Splice-Site Finder-like.

**Table 4:** Discrepant prediction between SpliceAI and MES/SSF.

Variant Region	Discrepant Prediction SpliceAI/MES/SSF	Number of Variants	Splice +/− Identified by cDNA and gDNA Seq ^1^
Exon	−/+	31	0/31
	+/−	9	3/6
Canonial splice-site	−/+	0	0/0
	+/−	3	3/0
Non-canonical intronic region	−/+	0	0/0
	+/−	2	2/0
Total	−/+	31	0/31
	+/−	14	8/6

^1^ Splice + denotes splicing alteration was confirmed by cDNA sequencing followed by gDNA sequencing, while Splice − denotes splicing alteration was not observed. Abbreviations: MES, MaxEntScan; SSF, Splice-Site Finder-like; cDNA, complementary DNA; gDNA, genomic DNA; Seq, sequencing analysis.

## Data Availability

The data that support the findings of this study are available in the supplementary material of this article. Any additional required data that support the findings of this study are available from the corresponding author upon reasonable request.

## Supplementary Materials

The following are available online at https://www.mdpi.com/article/10.3390/genes12091308/s1, Table S1: List of NF1 variants with their splicing effects and bioinformatic predictions. Table S2: PCR primer sets for whole NF1 genomic DNA sequence analysis. Table S3: PCR primer sets for whole NF1 complementary DNA sequence analysis.
