# Supplementary material for: Performance Evaluation of SpliceAI for the Prediction of Splicing of NF1 Variants
Source: Genes (Basel). 2021 Aug 25;12(9):1308. doi: 10.3390/genes12091308 (PMC8472818; doi:10.3390/genes12091308)
Supplement: Supplementary file 1 [file genes-12-01308-s001.zip › Table S2_S3.pdf]

Performance Evaluation of SpliceAI for the Prediction of Splicing of *NF1* Variants

Changhee Ha, Jong-Won Kim and Ja-Hyun Jang

Department of Laboratory Medicine and Genetics, Samsung Medical Center,

Sungkyunkwan University School of Medicine, Seoul, Republic of Korea

Table S2. PCR primer sets for whole *NF1* genomic DNA sequence analysis.

Fragement	Forward sequence	Reverse sequence	Product (bp)
1-RT	ctccacagaccctctccttg	aataaaaccccagaggcagaa	544
2-RT	aaaatgtcttgctgggcaac	gcaaaaccatccaccaagtc	498
3-RT	ggcattttggaactgggtag	gcaagaaacaaggcagtcaa	496
4-RT	tcattttcctacttgttcagtccat	gagctttggatctgcatgaat	497
5-RT	cacacccagcaatacgaatg	caggaacttctatctgcctgct	499
6-RT	gggagattagctcacaaatgc	tgtgttatagttgggcaagagg	500
7-RT	tgtggaaccctgacactgaa	gccagaattgcttctctgct	494
8-RT	ccatgtgagtggaggaggat	gatgttcagagctgccttca	494
9-RT	caaattgaagaataccatcagcaa	gcttccatcaattccacacc	489
10-RT	gaacatcaaaccaagcagca	tcacccatcattgtgaccag	499
11-RT	caaggatctccagacaagagcta	ctggttttcctcaaggctct	499
12-RT	cctggatcctttattacgaattg	aatcattgaaaggccgcata	500
13-RT	aatcctgccattgtctcacc	cctgatgcctagtcataaattcc	490
14-RT	tcttgcatacctgggtcctc	agcagccgctcatgatactt	465
15-RT	tttgttgtttttcctggcttt	cacactcctggtgcatgaag	466
16-RT	tgacatttattatgcttcggaaa	ccaaacaaaggtgtttcaattc	500
17-RT	ctgccaacaacaccctcttt	ttttgcacatccttccaataa	468
18-RT	ggtggcttgggatcaataaa	atgacctgtcccggtaactg	499
19-RT	tgaagagaccaagcaagttttg	tggctgtaatttggttagtgc	484
20-RT	tctcatgggcagataaagca	caatttctgtgtttgttaaccagagt	500
21-RT	gcaaatggatcattttgttgg	caacttcttagtgttggcctga	496
22-RT	taccttgcagccacctatcc	gcacaacaggaaagactttgg	466
23-RT	ggcgctgcttcttactgttc	aaaggttaaggttggcagtga	488
24-RT	tgccttgattgacacgtacc	gcagcattaaatttaggcaagg	496

Table S3. PCR primer sets for *NF1* complementary DNA sequence analysis.

Exon	Forward sequence	Reverse sequence	Product (bp)
1	cagaccctctccttgcctctt	ggatggagggtcggaggctg	439
2	tgtggttgatgcagttttcc	tccaaagtccacagaaaatca	368
3	tctgggaggtaaaatggaaga	ttgtctgtcaccaggtcagc	364
4	ttttgttctgtgtgtgtgtttga	acctaagcctgggaggtcaa	445
5	gtcttgaactcctggcctca	ttggtgttctagttcagcacaat	337
6	cgagttttgggtttatgttaggtg	ttcttcctgaaaatgaatgtgaaa	342
7	gtttcacattcattttcaggaag	gctccaaggaaaggaagagg	400
8	tgttgcccttgggtttttac	tggttccctgaaatactttgc	364
9	tgagttttagaggctgttaatttgc	tccatttaggctgatgaacaca	435
10	ttcttctggcagctggattt	aaggtcttgaaacgaacatcaa	381
11	tgctgttctttttggcttca	tatggtcccttcggtcaaga	362
12	atggtgtgtgtttgcatggt	ccaaagcagcaggaatagga	346
13	cttgtatgtatgtctggggaatg	ggatgagaacagagatctcatcaa	828
14	caaagtcctgggcttacagg	cagaaaccacacaccaaagg	372
15	tccctagaggtttgtgttcacc	caaaaccataaaacctttggaa	382
16	aatgccattcttatgtctggtt	tgagaacattgggaggaagg	326
17	ggagagtctcaaacaggaagaca	gcccaaagttccacaaagag	400
18	gtcttccacccttgactctcag	agatgccatgtgctttgagg	327
19	tttgggtggagcttatcagg	tcagctacagccaagtttcaa	331
20	ccctcaatttggaagcctct	tgtttactttactgagcgactcttg	372
21-1	tgtcatggaagaaatgttgga	agcattggatacagagcagga	481
21-2	gcagatacacctgtcagcaaatt	ccacaggcatagagccaaat	458
22	atttggctctatgcctgtgg	cacaccctagtttgtgtgcag	387
23	aaggtagccagaagttgtgtacg	tgctctcttttcatgtcctcct	393
24	aggtgtgtgtgtggcttcaa	tgacaacactaacttcccaaaca	375
25	gaggtttatttgaggggaagtg	ttcctatcctagtcctgtcatgg	301
26	tcgcgagagaggagagaaac	tggccttgctgaagtaatttt	400
27	caaaacctagagaactggcatgt	aacatctttcttctggctctgaaa	424
28	gcaagtggttgtcaactttgg	acaatcagccctgcacattc	351
29	tacagaatgtgcagggctga	gtccctggatctaaggcaaa	400
30	cgttgcacttggcttaatgt	ccacacaccatcagcagcta	334
31	ttgggtctcaacatttcttgc	cagtgaaggtcaaataggctga	392
32	tgcaaagtttgacctttgaact	caaaagcacataactgaaaacca	370
33	ttgggaaggttagaaacactacct	tgacctagagcaggaaaagaaaa	397
34	tgtgtgaacaagccctccat	ccaagaagatgcaaagtaaaaagc	376
35	tggtcctgaggtctttttgg	tcatatctaacaagtggcctggt	366
36	tggtgcatgttgccaaatta	ccctgctatatgctcccaag	399

37-1	ggagatttgtctcttctcttagcc	ttgctacctttgaggccagt	474
37-2	tgtttttcctggctttgctt	cgacagccttacgtgacattt	399
38-1	ctcccaaagtgctgggatta	agagtcgggctgtgacagtt	528
38-2	tgcttcggaaattgaagaaa	ccaacactgcataccttcca	381
39	tgatcatactttgtaacagaatcacaa	tcactggaaaattctgaaatgaaa	392
40	ttttctccaggcctgattct	ccaatgtggcaccagataaa	391
41	ttgattaggctgttccaatgaa	catgggactcaaagttttagca	352
42-1	tgctaaaactttgagtcccatgt	tggagaaaggaactggtaaaca	385
42-2	gctgtagctttggcttctgg	gcaacttggtgttagagcaca	593
43	gaggtttgatttagggaacatga	caaaatagcacaataaaccaatatttc	448
44	ttgcatggactgtgttattgg	tggttgcagggatggattat	355
45	gctggaccagtggacagaac	tttcattgacctcaaatttaaacg	391
46	tcctgaattcattccgagattc	tcctttagcactgatgagacca	423
47	ccccaaaagagaaaacatgg	gcaacaagaaaagatggaagag	332
48	tgttctgtggttttctgcagtc	gcctggcctagtttgcattt	415
49	cagggaagaagacctcagca	tgaccaagggaacacatttca	435
50	tgtgcacatttaacaggtactatgc	ctgctgcttgcctccattag	326
51	taggacagccacttggaagg	tgtttcaaggtcccattctca	447
52	tgggaacaaaaccctttgag	tggctggaaaataaacatgacttt	388
53	cagcattgtaaataggtagccaaa	tgtttgggtgagaagtagaagactg	431
54	ccccaccctcaaatttttatt	ctttgcaaccagtgcacatt	340
55	tgaagaaatgccccagaaag	agtcagtgcattctacaacagc	363
56	ggttttagttgctttgacactca	aatacacacaccccaacacca	391
57	tggcttcagatggggattta	gggaattcctaatgttggtgtc	346
58	atgtgtccccgttgttaagc	caaaccggatgggttcatta	366
